# Prostate cancer risk stratification via eNose urine odor analysis: a preliminary report

**DOI:** 10.3389/fonc.2024.1339796

**Published:** 2024-03-05

**Authors:** Gianluigi Taverna, Fabio Grizzi, Carmen Bax, Lorenzo Tidu, Matteo Zanoni, Paolo Vota, Cinzia Mazzieri, Maria Chiara Clementi, Giovanni Toia, Mohamed A. A. A. Hegazi, Beatrice Julia Lotesoriere, Rodolfo Hurle, Laura Capelli

**Affiliations:** ^1^ Department of Urology, Humanitas Mater Domini, Varese, Italy; ^2^ Department of Biomedical Sciences, Humanitas University, Milan, Italy; ^3^ Department of Immunology and Inflammation, IRCCS Humanitas Research Hospital, Milan, Italy; ^4^ Department of Chemistry, Materials and Chemical Engineering “Giulio Natta”, Politecnico di Milano, Milan, Italy; ^5^ “Vittorio Veneto” Division, Italian Ministry of Defenses, Firenze, Italy; ^6^ Department of Urology, IRCCS Humanitas Research Hospital, Rozzano, Milan, Italy

**Keywords:** prostate, cancer, risk-stratification, eNose, volatile organic compounds

## Abstract

**Introduction:**

Prostate cancer (PCa) is known for its highly diverse clinical behavior, ranging from low-risk, slow-growing tumors to aggressive and life-threatening forms. To avoid over-treatment of low-risk PCa patients, it would be very important prior to any therapeutic intervention to appropriately classify subjects based on tumor aggressiveness. Unfortunately, there is currently no reliable test available for this purpose. The aim of the present study was to evaluate the ability of risk stratification of PCa subjects using an electronic nose (eNose) detecting PCa-specific volatile organic compounds (VOCs) in urine samples.

**Methods:**

The study involved 120 participants who underwent diagnostic prostate biopsy followed by robot assisted radical prostatectomy (RARP). PCa risk was categorized as low, intermediate, or high based on the D’Amico risk classification and the pathological grade (PG) assessed after RARP. The eNose’s ability to categorize subjects for PCa risk stratification was evaluated based on accuracy and recall metrics.

**Results:**

The study population comprised 120 participants. When comparing eNose predictions with PG an accuracy of 79.2% (95%CI 70.8 – 86%) was found, while an accuracy of 74.2% (95%CI 65.4 – 81.7%) was found when compared to D’Amico risk classification system. Additionally, if compared low- versus -intermediate-/high-risk PCa, the eNose achieved an accuracy of 87.5% (95%CI 80.2-92.8%) based on PG or 90.8% (95%CI 84.2–95.3%) based on D’Amico risk classification. However, when using low-/-intermediate versus -high-risk PCa for PG, the accuracy was found to be 91.7% (95%CI 85.2-95.9%). Finally, an accuracy of 80.8% (95%CI72.6-87.4%) was found when compared with D’Amico risk classification.

**Discussion:**

The findings of this study indicate that eNose may represent a valid alternative not only for early and non-invasive diagnosis of PCa, but also to categorize patients based on tumor aggressiveness. Further studies including a wider sample population will be necessary to confirm the potential clinical impact of this new technology.

## 1 Introduction

After skin cancer, prostate cancer (PCa) is the most frequently diagnosed malignancy in American men (1). The risk of disease progression and adverse outcomes varies broadly based on clinicopathologic characteristics (2). Hence, accurate disease risk stratification is paramount to align the aggressiveness of management to the severity of disease. PCa is commonly stratified as low-, intermediate-, or high-risk, as first proposed by D’Amico et al. (3). Other risk stratification models have also been described (4–6). Prostate biopsy is a component of all such models. However, it is invasive and frequently underestimates pathological data due to grading and sampling errors, and borderline grades (7). An accurate and non-invasive approach would be a valuable enhancement to the currently available armamentarium. It has been demonstrated that highly trained dogs detect PCa-specific Volatile Organic Compounds (VOCs) in urines (8–10). To harness the potential of PCa-specific VOCs, an electronic nose (eNose), which mimics dogs’ capability to detect and recognize odors, was designed for widespread utilization in clinical settings (10–13), achieving a sensitivity of 85.2% and a specificity of 79.1% in diagnosing PCa (10). Recent studies indicate that analyzing urinary VOCs is a promising approach for discover new biomarkers for early cancer detection. Alterations in urinary VOCs have been linked to pathological conditions like tumors and infections. Various research has demonstrated the eNose ability to detect these changes in urine, successfully identifying various human neoplasia, such as prostate (10, 14), bladder (15), kidney (16), breast (17), lung (18) and colorectal (19) cancers.

In a recent study involving 252 participants, including 110 renal patients and 142 healthy individuals, urine samples were analyzed using a commercially available eNose (16). The device achieved a specificity of 89.4%, sensitivity of 71.8%, positive predictive value of 84.04%, and negative predictive value of 80.37% (16). Similarly, Filianoti et al. (14) applied the same technology to differentiate PCa patients from healthy controls. The study demonstrated a sensitivity of 82.7%, specificity of 88.5%, positive predictive value of 87.3%, and negative predictive value of 84.2%. These findings reinforce the potential of urine volatilome profiling with an eNose as a non-invasive diagnostic method. Matsumoto and colleagues (15) analyzed urine samples from 36 bladder cancer patients, 29 with urolithiasis, 10 with urinary tract infections, and 27 healthy volunteers. Using ROC analysis, they established optimal cut-off values for bladder cancer detection at θ=49 for healthy volunteers, θ=48 for urolithiasis, and θ=55 for urinary tract infections. Significant differences were noted between bladder cancer and other conditions at these thresholds (15). The diagnostic sensitivity was 61.4%, 45.6%, and 60.8%, while specificity was 52.8%, 68.4%, and 90.2%, respectively (15). These results support the use of the eNose as a cost-effective, non-invasive tool for distinguishing bladder cancer from benign conditions. Overall, the current findings confirm the eNose efficacy as a non-invasive, easy-to-use, and rapid response method for detecting disease-specific VOC patterns in both neoplastic and non-neoplastic diseases. It is important to emphasize that the eNose doesn’t furnish details about the chemical composition of samples; instead, it characterizes their overall VOC patterns. The aim of the present study was to evaluate the capability of risk stratification of PCa subjects using an eNose detecting PCa-specific VOCs in urine samples.

## 2 Materials and methods

### 2.1 Participants

A blind prospective cohort study on 120 consecutive PCa patients between January 2021 and September 2022 was carried out by the Urology Departments of Humanitas Mater Domini, Castellanza, Varese, Italy, the IRCCS Humanitas Research Hospital, Rozzano, Milan, Italy, and the Department of Chemistry, Materials and Chemical Engineering “Giulio Natta”, Politecnico di Milano. The study was approved by the ethical committee at IRCCS Humanitas Research Hospital (Approval no. CE-ICH260/11). Each participant was fully informed about the study and provided informed consent. The current study encompassed consecutive participants who underwent diagnostic prostate biopsy, followed by robot-assisted radical prostatectomy (RARP). We did not apply any exclusion criteria related to medical history, alcohol consumption, drug use, dietary habits, tobacco use, or other lifestyle factors.

**Table 1** presents the baseline demographics and clinical characteristics of patients, along with the (PCa) risk stratification based on D’Amico. Additionally, other widely utilized models in clinical practice, namely ISUP (5), CAPRA (4), and NCCN (6), were also employed to assess PCa risk and are detailed in the **Table 1**.

**Table 1:** Baseline epidemiological, clinical and histopathological characteristics of the study population.

	Subjects (*n=120*)
Age *[mean ± SD (range), years]*	61 ± 8 (48-77)
PSA *[mean ± SD (range), ng/mL]*	5.8 ± 4.37 (2-40)
Volume *[mean ± SD (range), mL]*	61.5 ± 20.88 (24-108)
PSA density *[mean ± SD (range), ng/mL^2^]*	0.1 ± 0.08 (0.03-0.63)
Clinical grade *n, (%)*	
3 + 3	30 (25)
3 + 4	41 (34)
4 + 4	35 (29)
4 + 5	12 (10)
5 + 4	2 (2)
Clinical Stage *n, (%)*	
T1b	2 (2)
T1c	73 (61)
T2	38 (32)
T2a	7 (5)
Pathological Grade *n, (%)*	
3 + 3	12 (10)
3 + 4	46 (38)
4 + 3	24 (20)
4 + 3+5	2 (2)
4 + 4	10 (8)
4 + 5	22 (18)
5 + 3	1 (1)
5 + 4	3 (3)
D’Amico Risk classification *n, (%)*	
Low-risk	30 (25)
Intermediate-risk	41 (34)
High-risk	49 (41)
ISUP n, (%)	
1	30 (25)
2	41 (34)
3	0 (0)
4	35 (29)
5	14 (12)
CAPRA Risk classification *n, (%)*	
Low-risk	31 (25)
Intermediate-risk	68 (57)
High-risk	21 (18)
NCCN Risk classification *n, (%)*	
Very-Low	1 (1)
Low	29 (24)
Intermediate Favorable	8 (7)
Intermediate Unfavorable	33 (27)
High	47 (39)
Very-High	2 (2)

To pursue the primary endpoint, the eNose results were compared with both the post-RARP pathological grade (PG) and the D’Amico risk-stratification score. Furthermore, the secondary endpoint of the study was to explore the agreement between eNose outcomes and ISUP, CAPRA, and NCCN scores. Additionally, to assess the potential for categorizing intermediate-risk PCa as either low-risk or high-risk based on eNose results, the study population was grouped into two categories: a) low-risk versus intermediate/high-risk PCa or b) low/intermediate-risk versus high-risk PCa.

### 2.2 Urine samples

Before the prostate fusion biopsy, two sterile urine containers were used to collect a spontaneous 30mL urine sample from each subject. The samples were then frozen at -20°C and subsequently transported under controlled temperature conditions and maintained at -20°C until the time of analysis.

### 2.3 eNose and experimental protocol

**Figure 1:**
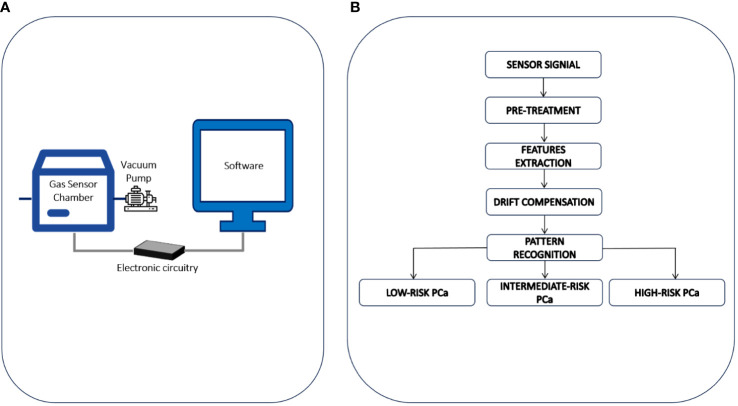
**(A)** The eNose involved in the study is a lab-scale prototype ideated and developed at the Department of Chemistry, Materials and Chemical Engineering of the Politecnico di Milano. It consists of: a) a sensor chamber equipped with 8 sensors: 6 n-type doped metal oxide semiconductor (MOS) sensors, working at 400°C and differing for active layers and 2 sensors for the continuous measurement of temperature and relative humidity; b) a vacuum pump for sucking gaseous urine samples from bag, thereby avoiding any contamination of the sample before the analysis; c) an electronic system for signal acquisition at a frequency of 1 Hz and pre-processing and d) a computer for signal processing. **(B)** Data processing procedure involved for PCa stratification by eNose.

The eNose is a lab-scale prototype ideated and developed at the Department of Chemistry, Materials and Chemical Engineering of the Politecnico di Milano (**Figure 1A**). It consists of: a) a sensor chamber equipped with 8 sensors: 6 n-type doped metal oxide semiconductor (MOS) sensors, working at 400°C and differing for active layers (i.e., TiO_2_, ZnO and SnO_2_ based sensors), and 2 sensors for the continuous measurement of temperature and relative humidity (RH); b) a vacuum pump for sucking gaseous urine samples from bag, thereby avoiding any contamination of the sample before the analysis; c) an electronic system for signal acquisition at a frequency of 1 Hz and pre-processing and d) a computer for signal processing. The experimental protocol for sample preparation and eNose analysis consists of four steps, as previously described (11, 12). In brief, a) *Thawing:* urine samples, stored at -20°C, are thawed in a water bath at about 40°C. b) *Urine headspace creation*: 10mL of liquid urine are put in a Nalophan^®^ bag filled of odorless air and conditioned at 60°C and 20% RH for 60 min to favor the enrichment of the gaseous phase with urine volatiles. c) *Urine headspace conditioning*: the gaseous phase is separated from the liquid and conditioned at 60°C and 20% RH for 90 min to reduce the moisture content and avoid water condensation in tubes during the analysis, and d) *eNose analysis*: the urine headspace is analyzed at a fixed concentration, recording the variations of resistance related to adsorption, and desorption, of VOCs on the sensors surface. This step takes a total of 80 minutes to complete.

### 2.4 Data processing

Sensors signals recorded during the analysis of urine headspaces have been processed according to the data processing procedure developed during the eNose training phase. The eNose has been trained from March 2016 to December 2020, using the urines collected from 329 subjects underwent RARP and accordingly to the PG categorized into 3 different risk groups: low GS = 6 (64 patients), intermediate GS =7 (131 patients), and high GS ≥ 8 (134 patients). The data processing procedure involved for PCa stratification by eNose is schematically illustrated in **Figure 1B**. Initially, the raw resistance curves obtained from gas sensors in the eNose array during urine headspace analyses underwent Standard Normal Variate (SNV) processing (20). This was done to offset baseline shifts observed in urine headspace analyses conducted on different days, likely stemming from external factors such as fluctuations in environmental temperature or humidity, which are not pertinent for sample classification (11). Then, numerical parameters, commonly referred as features, are extracted from sensor signals to build a training dataset. For each sample, a reference label was defined based on baseline epidemiological and clinical details, including the PG. Detailed information about the mathematical equations and the parameters used for features extraction have been previously reported (11, 12). After autoscaling and drift compensation the training dataset has been used to implement a pattern recognition model for PCa risk-stratification, based on Random-Forrest classifier (21). Subsequently, the eNose categorizes the examined sample, specifically identifying it as low-, intermediate-, or high-risk PCa. The validity of the pattern recognition model was later confirmed through an independent blind prospective cohort, which is the focus of the current study.

### 2.5 Statistical analysis

Baseline epidemiological and clinical characteristics were reported as mean ± standard deviation (SD) while frequencies were used for categorical variables. eNose risk-stratification performance was assessed in terms of accuracy, recall (22) and Cohen’s κ-coefficient. The accuracy of the eNose is determined by dividing the total number of correctly predicted outcomes by the total number of tests performed. Recall refer to the rate of correctly predicted outcomes per risk class, divided by the total number of tests conducted per risk class (22). We conducted all analyses using STATA16.1 (StataCorp. 2019. College Station, TX: StataCorp LLC) and applied a two-sided test, setting the level of statistical significance at p <0.05.

## 3 Results

Comparing the eNose with the PG after RARP, the eNose achieved an accuracy of 79% (95% confidence interval [CI]: 71 – 86%). When comparing the eNose with D’Amico risk-classification, the eNose exhibited an accuracy of 74% (95% CI: 65 – 82%). We also examined whether intermediate-risk PCa could be categorized as either low- or high-risk, assessing two scenarios: *a)* low- versus intermediate/high-risk, and *b)* low/intermediate- versus high-risk. When categorizing low-risk versus intermediate/high-risk PCa, the eNose demonstrated an accuracy of 88% (95% CI: 80 - 93%) when compared to the PG. Conversely, based on the D’Amico risk-classification, the eNose achieved an accuracy of 91% (95% CI: 84 - 95%).

**Figure 2:**
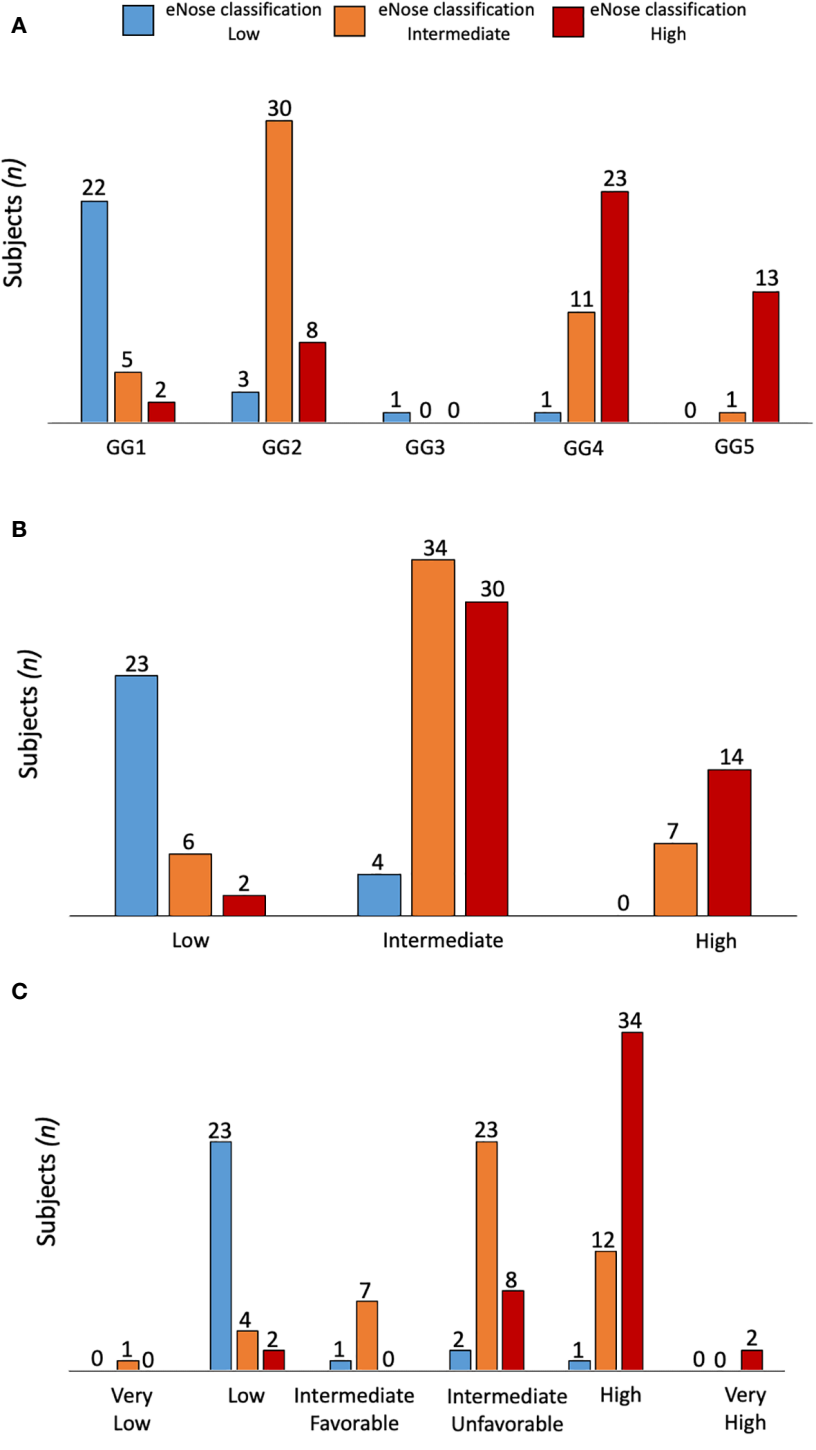
Comparison of eNose outcomes according to ISUP **(A)**, CAPRA **(B)** or NCCN **(C)** risk stratification models. The agreement between eNose and ISUP was 0.3 (95% CI: 0.15 - 0.51, p < 0.0003), while the agreement between eNose and CAPRA was 0.4 (95% CI: 0.19 - 0.55, p < 0.0001), and the comparison with NCCN yielded an agreement of 0.4 (95% CI: 0.23 - 0.59, p < 0.0001).

When grouping low/intermediate- versus high-risk PCa, the eNose showed an accuracy of 92% (95% CI: 85 - 96%) when compared with the PG, while an accuracy of 81% (95% CI: 73 - 87%) was found when compared with D’Amico risk-classification. **Table 2** summarizes all these findings, including the recall ratio. Cohen’s κ-coefficient revealed a substantial level of agreement between eNose and the PG of 0.7 (95% CI: 0.49 - 0.84, p < 0.0001), and between eNose and D’Amico risk-classification of 0.6 (95% CI: 0.42 - 0.78, p < 0.0001). Furthermore, there was an agreement between eNose outcomes and the other risk-stratification models (**Figure 2**). The agreement between eNose and ISUP was 0.3 (95% CI: 0.15 - 0.51, p < 0.0003), while the agreement between eNose and CAPRA was 0.4 (95% CI: 0.19 - 0.55, p < 0.0001), and the comparison with NCCN yielded an agreement of 0.4 (95% CI: 0.23 - 0.59, p < 0.0001).

**Table 2:** Accuracy and recall of eNose outcomes versus PG after RARP and D’Amico risk classification model.

eNose						
	Accuracy *(%, 95%CI)*	Recall *(%, 95%CI)*				
		L-risk	I-risk	H-risk	I-/H-risk	L-/I-risk
L- vs. I- vs H-risk						
PG	79% (95%CI 71 – 86%)	100% (95%CI 74-100%)	66% (95%CI 53-77%)	97% (95%CI 86-100%)	–	–
D’Amico Risk Classification	74% (95%CI 65 – 82%)	77% (95%CI 58–90%)	73% (95%CI 57–86%)	73% (95%CI 59–85%)	–	–
L- vs I-/H-risk						
PG	88% (95%CI 80-93%)	100% (95%CI 74-100%)	–	–	86% (95%CI 78-92%)	–
D’Amico Risk Classification	91% (95%CI 84–95%)	77% (95%CI 58–90%)	–	–	96% (95%CI 89–99%)	–
L-/I- vs H-risk						
PG	92% (95%CI 85-96%)	–	–	97% (95%CI 86-100%)	–	89% (95%CI 80-95%)
D’Amico Risk Classification	81% (95%CI 73-87%)	–	–	73% (95%CI 59-85%)	–	86% (95%CI 76-93%)

PG, Pathological grade; L-, Low-risk PCa; I-, Intermediate-risk PCa; H-, High-risk PCa.

## 4 Discussion

At present, it remains unclear which tool is the most effective for stratifying patients with PCa, but the risk of overdiagnosis and overtreatment persists in all approaches (7, 23, 24). The current scoring systems (3–6) rely on subjective features except for age, and all required a biopsy sampling. The Gleason score is a useful indicator of tumor aggressiveness, and a close correlation between biopsy and pathological specimens would be beneficial. However, biopsies frequently underestimate pathological data with discrepancies ranging from 27% to 46% (25). Prostate-specific antigen (PSA) has been found to have poor accuracy in predicting the severity of PCa. Despite improvements, multiple studies have also demonstrated significant variability in the quality of magnetic resonance imaging (MRI) scans and that patient-related factors can introduce errors (26, 27). Considering these challenges, a growing need exists for objective, accurate, non-invasive and cost-effective methods in selecting patients with PCa before an eventual diagnostic biopsy. In this study, we evaluated the eNose’s capability to stratify the risk of PCa. Our findings demonstrated that the eNose exhibits a high level of accuracy and agreement when compared to the PG, which is considered to closely represent the real state of the disease. Remarkably, the eNose also demonstrates an agreement with D’Amico and other risk-stratification models, possibly eliminating the need for invasive biopsy sampling. Additionally, in this current study, as no exclusion criteria were established regarding medical history, alcohol consumption, drugs, diet, tobacco, or other habits, it can be inferred that our results are not influenced by potential confounding variables. The accuracy of the technology is primarily dependent on the PCa-specific VOCs in urine samples, a finding that is corroborated by other studies (11, 28). Collectively, these initial findings indicate that eNose may have a significant role in classifying patients before resorting to costly and invasive procedures. This potential application could alleviate the financial strain on healthcare systems and reduce the risks for individuals under suspicion of having PCa. The tool could be further refined to serve as the primary screening method, potentially leading to a series of costly and invasive tests, contingent on the patient’s age and clinical condition. In fact, if the eNose indicates a potential presence of low-risk tumors, it could prompt a reevaluation of the entire subsequent diagnostic process. Additionally, the eNose may be effectively employed for longitudinal monitoring of patients over time, with minimal costs and in a non-invasive manner. After validation in a wider population and the refinement of pattern recognition models through additional targeted training, this device could be readily employed for detecting various neoplastic or non-neoplastic diseases. It is known that the methods used for feature extraction and training pattern recognition models play a critical role in enhancing the performance of the eNose system (29, 30). It is worth noting that a limitation of our study lies in the relatively small size of the investigated population. To enhance and confirm the efficacy of the eNose, a forthcoming multicenter study is needed, encompassing a larger and more diverse patient cohort. Although the eNose shows promising results, it is also crucial to acknowledge the existence of technical challenges. Furthermore, comparing the results of the eNose with MRI interpretations using the Prostate Imaging-Reporting and Data System (PI-RADS) presents an additional challenge. This comparison aims to determine whether there is a concordance between eNose outcomes and MRI, which remains the primary diagnostic test for men suspected of having PCa.

This preliminary study has shown that eNose represents a valuable tool not only to diagnose PCa but also to stratify its aggressiveness. The future direction will enhance the eNose risk-stratification model to provide tailored responses based on different risk-stratification models, enabling urologists to use it according to their personal practices.

## Data availability statement

The raw data supporting the conclusions of this article will be made available by the authors, without undue reservation.

## Ethics statement

The studies involving humans were approved by IRCCS Humanitas Research Hospital. The studies were conducted in accordance with the local legislation and institutional requirements. The participants provided their written informed consent to participate in this study.

## Author contributions

GTa: Conceptualization, Supervision, Writing – original draft, Writing – review & editing. FG: Data curation, Writing – original draft, Writing – review & editing. CB: Data curation, Formal analysis, Methodology, Writing – original draft, Writing – review & editing. LT: Writing – review & editing. MZ: Writing – review & editing. PV: Writing – review & editing. CM: Writing – review & editing. MC: Writing – review & editing. GTo: Writing – review & editing. MH: Data curation, Formal analysis, Methodology, Writing – review & editing. BL: Data curation, Formal analysis, Methodology, Writing – review & editing. RH: Writing – review & editing. LC: Conceptualization, Supervision, Writing – original draft, Writing – review & editing.
